# Neuropathic Pain and Rehabilitation: A Systematic Review of International Guidelines

**DOI:** 10.3390/diagnostics11010074

**Published:** 2021-01-05

**Authors:** Andrea Bernetti, Francesco Agostini, Alessandro de Sire, Massimiliano Mangone, Lucrezia Tognolo, Annalisa Di Cesare, Pierangela Ruiu, Teresa Paolucci, Marco Invernizzi, Marco Paoloni

**Affiliations:** 1 Department of Anatomical and Histological Sciences, Legal Medicine and Orthopedics, Sapienza University, 00185 Rome, Italy; francescoagostini.ff@gmail.com (F.A.); massimiliano.mangone@uniroma1.it (M.M.); annalisa.dicesare@uniroma1.it (A.D.C.); pierangela.ruiu@uniroma1.it (P.R.); marco.paoloni@uniroma1.it (M.P.); 2 Department of Health Sciences, University of Eastern Piedmont, 28100 Novara, Italy; alessandro.desire@gmail.com (A.d.S.); marco.invernizzi@med.uniupo.it (M.I.); 3 Rehabilitation Unit, “Mons. L. Novarese” Hospital, 13040 Moncrivello, Italy; 4 Department of Neurosciences, University of Padua, 35121 Padua, Italy; lucrezia.tognolo@unipd.it; 5 Department of Medical and Oral Sciences and Biotechnologies, University of G. D’Annunzio of Chieti-Pescara, 66100 Chieti, Italy; teresapaolucci@hotmail.com

**Keywords:** neuropathic pain, rehabilitation, guidelines, recommendation, instrumental physical therapy

## Abstract

Background: Neuropathic pain is an injury or disease of the central and/or peripheral somatosensory nervous system, and it has a significant impact on quality of life, especially since it is often refractory to treatment. Rehabilitative intervention is considered in various guidelines on neuropathic pain treatment, although not in an organic nor detailed way. The aim of this systematic review was to analyze the most indicated therapeutic strategies, providing rehabilitative recommendations in the management of neuropathic pain. Methods: A systematic review was performed according to PRISMA guidelines. The scientific search, carried out until July 2020, considered guidelines in English language of the last thirteen years. Results: Six guidelines were analyzed, from which emerges that a multidisciplinary approach, comprehensive of pharmacologic and nonpharmacologic interventions, should drive neuropathic pain management. A relevant role in non-pharmacological intervention is played by rehabilitation, through an adequate tailored rehabilitation program and physical therapies. Conclusion: This analysis highlights the importance of rehabilitation but also the lack of evidence on various rehabilitative practices. Arises hence the need for further studies in this field to better define a rehabilitative treatment strategy.

## 1. Introduction

According to the International Association for the Study of Pain (IASP) pain is defined as: “An unpleasant sensory and emotional experience associated with actual or potential tissue damage, or described in terms of such damage” [1]. Pain is an extremely complex phenomenon, which is composed of a perceptive and an emotional component. The perceptive component, called nociception, constitutes the neurobiological basis of pain and is achieved through the reception and transport of potentially harmful stimuli to the central nervous system, while the real individual experience of pain, the so-called emotional component, is the psychic state linked to the perception of an unpleasant sensation. Thus, the whole experience of pain is the result of affective and cognitive dimensions, experiences, psychic structure, and sociocultural factors.

Pain in the acute setting is a physiological defense system, a vital/existential symptom essential to avoid harm. It shifts to a pathological phenomenon in the chronic setting where it loses its protective function and becomes a self-perpetrating pathological symptom [2]. Among the different types of pain, neuropathic pain is defined as an injury or disease of the central and/or the peripheral somatosensory nervous system. [3] It affects millions of people worldwide, [4] with a prevalence in the general population in Europe estimated between 6.9 and 10% [5].

Neuropathic pain is generally chronic and severe, [4] affecting patients’ psychosocial and economic wellbeing and having a significant impact on quality of life [6]. Moreover, it is often resistant to treatment and associated with poor patients’ therapy satisfaction [7]. Thus, the management of this condition is extremely challenging for clinicians [4].

Many people with severe neuropathic pain conditions are significantly disabled with moderate or severe pain for many years. Chronic pain conditions comprised five of the 11 high-ranking conditions for years lived with disabilities in 2010 and are responsible for a notable loss in quality of life and employment and rising healthcare costs [8,9,10].

The are several pathophysiological mechanisms underpinning both central and peripheral neuropathic pain arousal including mechanical-compressive, traumatic, metabolic, endogenous, exogenous, neurotoxic, viral, inflammatory, and vascular causes. Due to this complex etiopathogenesis, neuropathic pain should be distinguished from other types of pain through a prompt and accurate diagnosis supported by the finding of typical symptoms like burning, tingling, or electric shock-like pain, that can be triggered even by a subtle tactile stimulation (allodynia) [11]. An accurate anamnesis and physical examination of the patient, along with diagnostic procedures, is crucial to properly define the clinical picture and represent the starting point to set a correct therapeutic strategy. Patient history allows to characterize pain, identify associated symptoms, determine the presence of comorbidities and, not less important, the impact on social, work-related, and economic sphere [12]. Moreover, neuropathic pain should be referred to a skin area with a corresponding peripheral or central nervous injury/disease with other signs and symptoms localized in this area. Thus, a careful objective examination is mandatory in order to assess the painful area, to identify any alteration of color, texture, temperature, and to perform a comparison with the non-painful adjacent area [13,14,15]. Lastly, in order to perform a correct differential diagnosis of neuropathic pain, it is useful to evaluate the exact quality of somatosensory, vegetative, and motor abnormalities both in the affected area, as in contiguous areas to sensory deficit. Several clinical and instrumental tools, such as functional evaluation scales, electromyography (EMG), and nerve conduction studies (NCSs), could help the clinician to characterize the presence and quality of neuropathic pain. In particular, in patients with neurologic signs or symptoms suggesting peripheral neuropathy, electromyography, and nerve conduction studies can be performed to better define the suspected diagnosis [6]; they investigate the function and integrity of large, myelinated A beta nerve fibers and are useful to define if there is sensory or motor involvement, to localize the process, to determine the severity of neuropathy and whether it is due to axonal loss, demyelination, or both. Nevertheless, EMG and NCSs do not assess small nerve fibers (C and small myelinated A delta fibers), therefore normal EMG findings do not exclude small-fiber peripheral neuropathy [16].

Diagnostic nerve blocks may be indicated to further narrow down the source of the pain. Furthermore, magnetic resonance imaging (MRI) can be employed to detect central lesions, while skin biopsy can be used to identify small fiber neuropathy [6]. Nerve biopsy may be useful to detect possible inflammatory-mediated, infiltrative, and some infectious neuropathies [16].

For a complete diagnostic process, functional evaluation and diagnosis of the patient result essential. Functional assessment in rehabilitation is complementary to all other evaluations and has the main goal to define, predict, and modify disability. Function, severely impaired in most patients with neuropathic pain, is usually assessed using disability scales, which explore function loss on various settings and its evolution over time. Disability scales employed are both bio-psychometric and functional. Functional evaluation and diagnosis in rehabilitation also involve motion analysis, including various measures, instruments, or devices to study gait and posture in patients with neuropathic pain. For example, quantitative gait analysis is a viable tool for Charcot-Marie-Tooth (CMT) functional gait assessment, even differentiating gait functional changes between CMT1 and CMT2 types [17]; it could also reveal alterations (as increased hip and knee flexion during early stance, prolonged hip extension moment in midstance, increased ankle dorsiflexion and power absorption throughout late stance and delayed and truncated final push-off) and consequent compensatory mechanisms in diabetic neuropathy [18]. Dynamic plantar pressure analysis highlights restrictions in subtalar and metatarsophalangeal joints and gait alterations in patients with diabetic neuropathy. It can evidence pressure distributions changes (of great clinical significance for ulcer prevention) even before the onset of deformity and degeneration of plantar soft tissue; patients with diabetic neuropathy could present lateral shift of peak pressure during walking and increased peak plantar pressure at medial heel region [19].

It is noteworthy, that despite the use of screening tools, about 10–20% of patients with neuropathic pain are not correctly identified, highlighting the crucial role of a prompt and effective clinical evaluation in this condition [20,21]. The most commonly used evaluation scales in the clinical setting are: Leeds Assessment of Neuropathic Symptoms and Signs (LANSS); Neuropathic Pain Questionnaire (NPQ); Douleur Neuropathique en 4 questions (DN4).

The pharmacologic treatment of neuropathic pain commonly relies on various pharmacological interventions, including tricyclic antidepressants, SSNRI, calcium-channel anticonvulsants, and opioids [3]. Along with these pharmacological treatments, rehabilitative interventions, including therapeutic exercise and physical therapies, play a pivotal role in the complex management of neuropathic pain. Thus, in this scenario, Physical Medicine and Rehabilitation specialists play a key role in both the diagnostic and therapeutic phases of this condition. Considering the psychosocial limitations, undermining the independence and the level of integration into the social context of the patient affected by neuropathic pain, the complex and multidisciplinary rehabilitative interventions should be aimed at not only to pain management, but also to guarantee the maximum achievable degree of participation in social life and quality of life implementation, directing towards an integrated therapeutic program. Albeit it is well known that rehabilitative intervention has a key role for neuropathic pain treatment, detailed and structured indications about the most effective interventions and a review of the evidence about this topic are still missing.

The contribution of Physical Medicine and Rehabilitation specialist should be aimed not only to manage neuropathic pain in the strict sense, but also to guarantee the maximum achievable degree of participation in social life through the rehabilitative intervention. Such orientation is needed since neuropathic pain results in psychosocial limitations for the patients, limiting their independence and their level of integration into the social context. To overcome these issues, it is required a global therapeutic strategy operating on various dimensions, physical and functional, psychological and social, which together influence the disease evolution. The integrated action on multiple variables surely requires the involvement of a multidisciplinary team with a holistic vision, in order to provide complete care.

In light of these considerations, the aim of this study is to perform a systematic review of the guidelines published over the last thirteen years about rehabilitative interventions in patients affected with neuropathic pain.

## 2. Materials and Methods

A systematic review was performed according to PRISMA guidelines [22].

A scientific literature search using the following databases: PubMed, PEDro, Cochrane, and Google Scholar was performed, from 1 October 2019 to 1 July 2020. The search was performed using the following Mesh terms: (rehabilitation AND neuropathic pain) OR (guidelines AND neuropathic pain) OR (treatment AND neuropathic pain) OR (instrumental physical therapy AND neuropathic pain) OR (physical therapy AND neuropathic pain) OR (management AND neuropathic pain). Specific databases for guidelines, such as guideline.gov, and the websites of the main international scientific societies that deal with the topic, have also been consulted. The inclusion criteria were the following: practice guidelines addressing the topic of rehabilitative management of patients suffering from neuropathic pain; evidence obtained in human subjects; guidelines in the English language; publication dates (13 years). The exclusion criteria were the following: full text not available; guidelines lacking rehabilitative interventions recommendations for neuropathic pain management and/or not providing evidence-based support. The scientific search, carried out until July 2020, reported the presence of recent guidelines related to neuropathic pain treatment. Three authors independently performed all the search and removed duplicate records. Three authors performed an independent data extraction and a narrative synthesis of the selected guidelines was performed. We evaluated inter-rater reliability, evaluating the degree of agreement between the choices made by three independent judges, using the Cohen’s *k*.

### PICO

Guidelines were considered that included patients, men and women of all ages, with mild to severe neuropathic pain, with different etiologies, and studying the effects of rehabilitation treatment on pain and functioning. The rehabilitation treatments considered were therapeutic exercise, stretching, massage, physiotherapy, behavioral therapy, multidisciplinary approach, and instrumental physical therapy. The recommendations expressed were extrapolated and compared in order to highlight consensus and discrepancies between them. The main findings considered were pain and functioning [23].

## 3. Results

**Figure 1:**
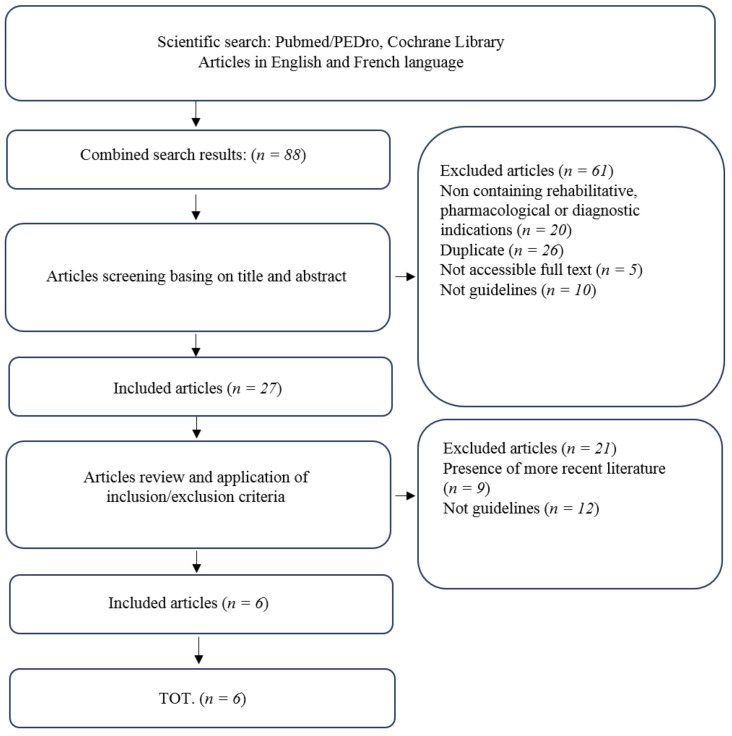
Flow Chart: PRISMA 2009 Flow Diagram.

Six guidelines matched the inclusion criteria (% of agreement: 84.61538461538461%; Mean Cohen’s *k* value: 0.6388888888888888—Substantial agreement) (Figure 1). The main characteristics of the included guidelines are resumed in Table 1. In the presence of articles published by the same working group, those most recently published have been taken into consideration.

Cruccu et al. [24] focused on neurostimulation therapy for neuropathic pain, providing guidelines about the various available techniques. Transcutaneous electrical nerve stimulation (TENS) may be superior to placebo and is suitable as preliminary or add-on therapy for neuropathic pain management. Demarin et al. [25], suggested the importance of a multidisciplinary approach for neuropathic pain treatment, including both interventional and non-interventional therapies (pharmacological, psychological, and physical therapy), highlighting the crucial role exerted by the latter in the overall management of this complex condition. It has to be noted that, among the non-pharmacological interventions, only TENS in painful diabetic neuropathy was superior to placebo. Martinez et al. [26], recommended transcutaneous electrical nerve stimulation, spinal cord stimulation and cognitive behavioral therapy as effective non-pharmacologic interventions to treat neuropathic pain. Moreover, they further suggested the use of acupuncture to treat post-herpetic neuralgia and recommended TENS for localized peripheral neuropathic pain treatment and spinal cord stimulation for chronic postoperative lumbar back pain with predominant radiculopathy. Bril et al. in their guidelines [27] focused on painful diabetic neuropathy, suggesting both pharmacological and non-pharmacological treatments to reduce pain and improve the quality of life of these patients. However, in these guidelines, the authors recommended only percutaneous nerve stimulation for painful diabetic neuropathy management. Chetty et al. [28] stressed the crucial role of the multidisciplinary approach in neuropathic pain management. The authors recommended, among non-pharmacologic treatment, a combination of psychotherapy and TENS associated with an adequate physiotherapy. Moreover, they further recommend deep brain stimulation for neuropathic pain refractory to other pharmacological and non-pharmacological interventions. Acevedo et al. [12] guidelines underlined how non-pharmacologic management and multimodal pharmacologic therapy, acting on different targets, could provide better therapeutic success than single interventions. Moreover, the authors suggested interdisciplinary management of neuropathic pain, including rehabilitative interventions.

## 4. Discussion

In this paper, we performed a systematic review of the guidelines published over the last thirteen years regarding rehabilitative interventions in neuropathic pain in order to clarify their role. Although some interventions like TENS therapy, physiotherapy, and psychological interventions were recommended, the key stone of neuropathic pain management lays in the implementation of multidisciplinary complex interventions focusing on all the different aspects affected by this chronic and extremely disabling pathological condition. Moreover, it is important to underline the lack of papers addressing the role of rehabilitation in patients affected by neuropathic pain, focusing on single interventions only aimed at treating neuropathic pain.

Among rehabilitative interventions, physical therapies commonly play a pivotal role in reducing pain. In this systematic review, we evidenced that several studies suggest TENS for neuropathic pain [7,24,25,26,28]. More in detail, Martinez et al. recommend TENS for localized peripheral neuropathic pain and Demarin et al. [25] for painful diabetic neuropathy. Cruccu et al. [24] give a level C recommendation to TENS, which may be better than placebo; they consider its employment as preliminary treatment or additional therapy. Moreover, TENS might exert is action also in association with physiotherapy and psychotherapy for neuropathic pain management [7].

Several International Guidelines [12,28] recommend physiotherapy, although not providing clear indications for intervention modalities, for treating neuropathic pain. In this context, Cohen et al. [28] claim that, among complementary and alternative interventions, the strongest evidence is showed by therapeutic exercise for neck pain management. A recommendation has been detected for percutaneous nerve stimulation in painful diabetic neuropathy by Bril et al. [27]. Furthermore, spinal cord stimulation has been recommended [24,26] to treat chronic postoperative lumbar back pain with predominant radiculalgia; it is further suggested by Cruccu et al. [24] for complex regional pain syndrome. In this regard, Zilliox et al. [7] suggests spinal cord stimulation for failed back surgery syndrome, complex regional pain syndrome and for diabetic neuropathy. Moreover, it has been suggested [28] that deep brain stimulation should be reserved for neuropathic pain unable to be treated with pharmacological and companion treatments; in addition, Cruccu et al. [24] suggest performing this procedure only in experienced centers.

Nevertheless, there is a broad agreement in the scientific literature to give proper attention and not underestimate psychological issues. They are frequently encountered, confirming the repercussion on psychosocial sphere and quality of life by a condition, which is chronic, disabling, and difficult to manage. Several authors [24,25] recommend psychological intervention in the management of neuropathic pain. Additionally, Acevedo et al. [12] recommend carrying out a psychosocial assessment at the primary level of care to these patients. Even Martinez et al. [26] and Chetty et al. [28] support psychotherapy, suggesting cognitive behavioral therapy.

The greatest consensus is on the recommendation of a multidisciplinary intervention for neuropathic pain, involving pharmacological therapy in association with non-pharmacological one (psychotherapy, physical therapies, and physiotherapy). This is observed on Demarin et al. [25], Chetty et al. [28] and Acevedo et al. [12] studies. In this regard, even recent reviews have been published, whose authors (Binder et al. [3], Cohen et al. [29], Deng et al. [4] and Zilliox et al. [7]) are strongly in favor of the multidisciplinary approach, a key element essential to target different aspects in neuropathic pain treatment.

Taken together, we showed that TENS is broadly recommended in the management of patients with neuropathic pain, both performed as a preliminary treatment or in association with other interventions, as a component of a multimodal approach. This technique presents some favorable features considering that it is a non-invasive and safe treatment, with relatively few contraindications. TENS is also inexpensive, and this aspect should be taken in consideration since neuropathic pain is a condition that commonly entails a considerable cost for both the healthcare system but especially for the patients. A broad agreement on physiotherapy (alone or as complementary treatment) in the management of neuropathic pain emerges from the recent scientific literature; it might be provided for patients with neuropathic pain. Physiotherapy is necessary to prevent or reverse changes in trophism, disuse atrophy, subsequent contractures, and deformities, preventing ankylosis and deconditioning in general. Psychological assessment, followed by an appropriated psychotherapy is also suggested considering that neuropathic pain frequently results in various psychological sequelae (i.e., anxiety, depression, and sleep disturbances), which further worsen quality of life of the patient.

A combination of an appropriated physiotherapy program alongside psychotherapy, physical therapy, and pharmacological treatment, should be provided for neuropathic pain patients, which include also physical, psychological, and social issues.

Indeed, neuropathic pain presents different severe implications not only related to pain itself, but also to the consequent impairment of function that this entails. Therefore, the intervention of a specialist focused on functional impairment management and recovery is required. This need can be addressed by the Physical Medicine and Rehabilitation specialist, which deals with functional issues responsible for the patient’s disability.

Severe neuropathic pain is difficult to treat effectively, with only a minority of people experiencing a clinically relevant benefit from any single intervention. A multidisciplinary approach is now advocated, combining pharmacological interventions with physical or cognitive interventions (or both). The evidence for interventional management is very weak or non-existent. In those patients with very severe or treatment-refractory pain, surgical options should be considered [8,29].

Physical Medicine and Rehabilitative specialists should set-up and implement a multi-professional and inter-disciplinary intervention leveraging on a strict collaboration with various healthcare professionals involved in the patient management, in order to ensure the best management of pain. Moreover, it is mandatory to act through an integrated and holistic approach on the various dimensions influenced by neuropathic pain: physical, functional, affective, emotional, social, relational and work-related. A global and customized intervention with necessary active involvement and contribution of the patient in order to maximize the impact on his health-related quality of life [30,31].

We are aware that this study is not free from limitations: first, the absence of assessment of the quality and reporting of practice guidelines by AGREE-II instrument and review protocol have not been pre-registered; second, the heterogenicity and the complexity of neuropathic pain, in its etiology, signs, and symptoms and thus diagnosis; third, a significant limitation is represented by the low to moderate quality of evidence on neuropathic pain intervention modalities in literature, especially regarding rehabilitative ones.

However, to the best of our knowledge, the main strength of this study is that it addresses neuropathic pain treatment from a rehabilitative point of view for the first time in literature. Although evidence on rehabilitative intervention are not conspicuous, in consequence of the serious limitation of function characterizing the majority of patients with this pathology, the present review was needed to formulate summarized indications of the recommendations included in international guidelines.

## 5. Conclusions

Taken together, the studies included in the present systematic review showed how a multidisciplinary approach, comprehensive of pharmacologic and nonpharmacologic interventions, should drive neuropathic pain management. 

In this context of interdisciplinary treatment, a relevant role in non-pharmacological intervention is surely played by rehabilitation which should be implemented since the early phase of neuropathic pain management, acting in synergy with other interventions in order to achieve the best outcome.

This systematic review highlights the crucial role of rehabilitation in the framework of a multidisciplinary management of neuropathic pain, but also the lack of evidence on various rehabilitative practices to treat this challenging condition. Therefore, further studies are warranted to define the most appropriate rehabilitation interventions in patients affected by neuropathic pain.

## Figures and Tables

**Table 1:** Guidelines included in the systematic review.

No	Title, Year	Authors	Journal	Main Findings
1	EFNS guidelines on neurostimulation therapy for neuropathic pain, 2007.	Cruccu, G.; et al [24]	Eur J Neurol	TENS may be superior to placebo and is suitable as preliminary or add-on therapy.
2	Recommendations for neuropathic pain treatment, 2008.	Demarin, V.; et al [25]	Acta Clin Croat	Multidisciplinary intervention is suggested. TENS is superior to placebo in painful diabetic neuropathy.
3	Chronic neuropathic pain: diagnosis, evaluation and treatment in outpatient services. Guidelines for clinical practice of the French Society for the Study and Treatment of Pain, 2010.	Martinez, V.; et al [26]	Douleur analg	TENS, spinal cord stimulation and cognitive behavioral therapy are suggested for neuropathic pain treatment.
4	Evidence-based guideline: Treatment of painful diabetic neuropathy: report of the American Academy of Neurology, the American Association of Neuromuscular and Electrodiagnostic Medicine, and the American Academy of Physical Medicine and Rehabilitation, 2011.	Bril, V.; et al [27]	Neurology	Percutaneous nerve stimulation is recommended for painful diabetic neuropathy.
5	Clinical practice guidelines for management of neuropathic pain: expert panel recommendations for South Africa, 2012.	Chetty, S.; et al [28]	S Afr Med J	A combination of psychotherapy, TENS and physiotherapy is suggested for neuropathic pain treatment in a context of multidisciplinary approach.
6	Guidelines for the diagnosis and management of neuropathic pain: consensus of a group of Latin American experts, 2009.	Acevedo, J.C.; et al [12]	J Pain Palliat Care Pharmacother	An interdisciplinary management including rehabilitative intervention is suggested for neuropathic pain treatment.
